# The Use of Internet of Things (IoT) Technology to Promote Children's Oral Health: A Scoping Review

**DOI:** 10.1055/s-0043-1776116

**Published:** 2024-01-10

**Authors:** Moh Khafid, Taufan Bramantoro, Ninuk Hariyani, Dini Setyowati, Retno Palupi, Putu Aditya Ferdian Ariawantara, Dyah Nawang Palupi Pratamawari, Pindobilowo Pindobilowo, Nor Azlida Mohd Nor

**Affiliations:** 1 Faculty of Dental Medicine, Universitas Airlangga, Surabaya, Indonesia; 2 Faculty of Dentistry, Institut Ilmu Kesehatan Bhakti Wiyata, Kediri, Indonesia; 3 Department of Dental Public Health, Faculty of Dental Medicine, Universitas Airlangga, Surabaya, Indonesia; 4 Department of Public Administration, Faculty of Social and Political Sciences, Universitas Airlangga, Surabaya, Indonesia; 5 Student of Doctoral Program, Faculty of Dental Medicine, Universitas Airlangga, Surabaya, Indonesia; 6 Department of Community Oral Health & Clinical Prevention, Faculty of Dentistry, University of Malaya, Malaysia

**Keywords:** Internet of Things (IoT), oral health promotion, telemedicine, dental health

## Abstract

Dental treatments and oral health promotion are now more mobile and versatile thanks to the Internet of Things (IoT)-based healthcare services. This scoping review aims to compile the available data and outline the aims, design, assessment procedures, efficacy, advantages, and disadvantages of the implementation of IoT to improve children's oral health. Articles for this review were gathered from PubMed, Scopus, and Ebscohost databases to identify and construct the keywords and primary research topic. The selected studies were published between 2000 and 2022 and focused on children aged 1 to 18 and/or parents/caregivers of children who received oral health promotion and/or dental disease preventive treatments utilizing the IoT. Each study topic required data extraction. A total of nine papers were included in this review. Two of the nine publications were quasi-experimental, while the remaining six papers were randomized control trials. The nine papers considered in this appraisal have a range of interventions and follow-up periods. Mobile-Health (m-Health), home healthcare, hospital/clinical management, and electronic-Health applications (e-Health) are the most common IoT architecture used as interventions. Three studies assessed oral health knowledge and behavior scores, whereas the bulk of studies (6/7) used m-Health treatments focusing on dental plaque buildup as well as gingival health evaluation to assess oral hygiene. IoT is one of the mediums or instruments that might be used to encourage children's dental health. The studies suggest that the use of IoT could help in improving oral hygiene and oral health, which can further improve children's oral health.

## Introduction

Dental caries denotes an infectious disease that is influenced by several variables. Despite being preventable, it is a significant global public health concern. According to the Global Burden of Disease Study in 2015, dental caries is also the most common disease, affecting 1.3 billion permanent teeth and 560 million primary teeth. 1 2 Dental caries poses as one of noncommunicable diseases with the greatest widespread, affecting all ages, particularly youngsters, having an adverse effect on their general health through nutritional or inflammatory pathways, well-being, social relationships, and economic standing. 3 A complete preventative strategy 4 is required as it cannot be properly handled by a single preventive measure. Thus, in this age of sophisticated digital technology, the Internet of Things (IoT)-based intelligent services are vital since they may offer adequate solutions in a management system or service within many industries, including healthcare.

IoT refers to a wide range of modern technologies that may link the physical world (which includes items) and the digital one (which refers to information). The IoT is a network of physically interconnected items in the early modern period. This network that is connected through the Internet provides information to users. 5 6 “A system of interconnected computing devices, machines and digital machineries, objects, animals, or people over a network with unique identifiers and capable of transferring data without the need for human-to-human or human-to-computer communication,” is how the World Health Organization (WHO) defines the IoT. 7 IoT is considered as an ecosystem that contains smart objects equipped with sensors, networking and processing technologies integrating and working together to provide an environment in which smart services are taken to the end users. IoT is the biggest emerging trend in technology that has launched an unprecedented information revolution. Smartphones are parts of IoT smart devices, which play an important role in the growth of IoT devices. Using smart devices give so much benefits, such as better autonomy, better communication, and facilitating knowledge sharing that can increase productivity. 8

The IoT is a term used to describe any smart device that can monitor health data and connect to the Internet to perform different telehealth services, including telemonitoring, aged care supervision, teleconsultations, and robotically-assisted surgery. 9 10 IoT devices share the same mobile network infrastructure with other devices, most are smartphones, but perform different communication mechanisms, accomplish different functions, and confront different security risks. IoT in healthcare has the potential to result in the creation of a variety of medical applications, such as remote health monitoring that might enable caregivers to stream patients' health conditions in real-time and compliance with treatment or medication at home by healthcare professionals. 5 11

The WHO recommended digital health interventions using IoT for health education, health promotion communication based on the previously known health status or medical history, behavior-change communication, preventive and wellness services announcement, and publication of health events toward a target population based on their demographic characteristics. 1 The WHO has prioritized disease prevention and oral health promotion as well as the promotion of a good dental lifestyle, particularly for children and parents/caregivers. 12 13 Previous research revealed that parental and child-oriented initiatives to promote oral health have been shown to lower childhood caries rates, and parents of young children are aware of the financial benefits of preventing rather than treating dental problems. 14 15 16

Globally, the number of mobile and Internet users has grown rapidly in recent years, improving the way to educate and communicate with patients. 17 Since applications are available on smartphones, no other devices would be required to access them. Besides, personalized and precise reminders and instructions for health behaviors are available, complemented with real-time evaluation and feedback. Those features make IoT easy to use. 18

Another use of IoT can be found in hospital administration, through the use of a Radio Frequency Identification-based system for precise patient identification and prescriptions. 19 This approach has the potential to significantly reduce medication and human error. Furthermore, IoT technology can also be used in hospital management systems for early diagnosis and management of post-treatment complications, as well as providing appropriate interventions. 10 Another study observed the use of IoT for automated text messaging in a university dental clinic to remind and motivate the patient in practicing excellent oral hygiene during treatment. 20

More details are required on the use of IoT technology systems to promote children's dental health. A scoping analysis of published studies was conducted to compile evidence related to oral health promotion utilizing IoT technology to fill the knowledge gap. This review aimed to focus on recently published studies and synthesize information on the utilization of IoT technology for oral health promotion in children. These were done to deliver recommendations on IoT system utilization to prevent dental diseases and promote dental health, specifically in children.

## Methods

A scoping review denotes a measure of reviewing literature aiming to map, scope, and understand the state of the existing literature, also synthesizing knowledge on a topic of interest with regard to its volume, range, and characteristics. 21 22 23 As such, a scoping review is a proper option for exploring the utilization of IoT technology to promote children's oral health. The procedure of the current scoping review was adapted from Preferred Reporting Items for Systematic Reviews and Meta-Analyses (PRISMA) guidelines, while the framework was based on the five-stage methodological scheme proposed by Arksey and O'Malley. 21

### Data Sources and Search Strategy

The literature identification process took place from January to June 2022. Three indexed databases were selected: PubMed, Scopus, and Ebscohost. The search strategy was refined based on keywords considered relevant to the topic and is presented in Supplementary Appendix 1 (available in the online version).

Further reference tracking was done through the Google search engine and links to the related articles suggested by the database were added to this identification.

### Article Qualification

The subsequent criteria were applied to select studies that were eligible for inclusion: (1) involves child-parent dyads with children aged 1 to 18 years; (2) uses IoT- related media in oral health promotion and/or dental disease prevention interventions; (3) papers with a randomized controlled trial or quasi-experimental study design; (4) assesses dental caries and changes in oral hygiene, oral health-related behaviors, knowledge, attitude, or practice as outcome measures. The articles should be written in English and published between 2000 and 2022. The language restriction was considered due to limited time and resources for translation, while the date restriction was considered as the date when IoT started to be widely used.

### Article Selection, Data Extraction, and Synthesis

**Fig. 1:**
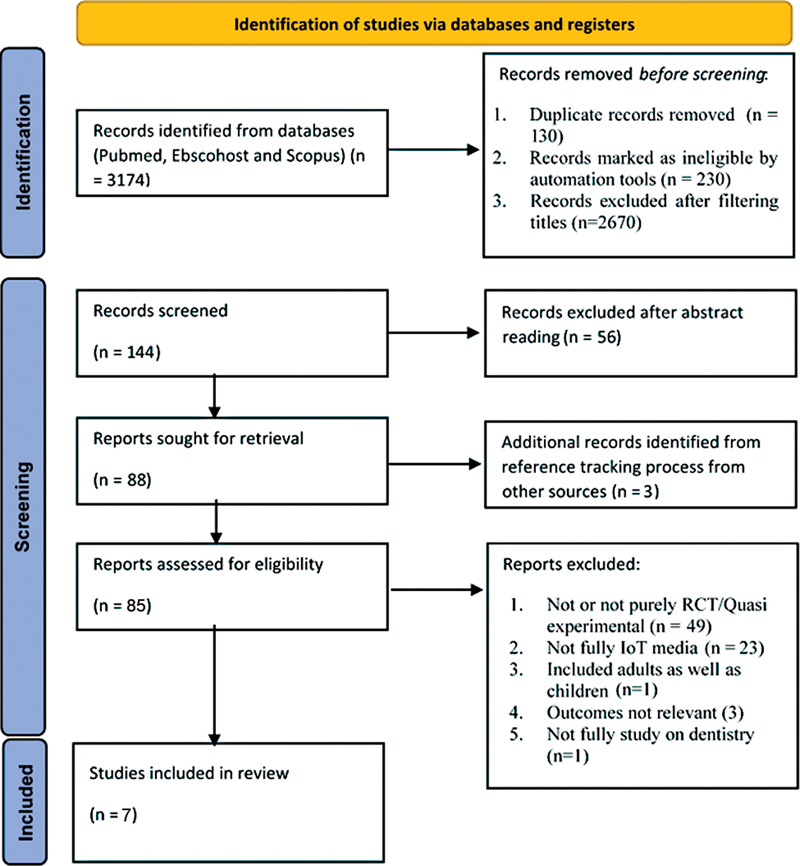
Diagram of study selection process. IoT, Internet of Things; RCT, randomized controlled trial.

There were two steps of screening during the article selection. The first step was examining the relevancy of the titles and abstracts performed by two reviewers independently. Thereafter, the reviewers reviewed the full-text articles. The third reviewer participated to resolve any disagreements and possible conflicting decisions. The procedure of article selection is described in Fig. 1 . There were two reviewers who completed the data extraction. The data from each included article were extracted based on authors, publication date, country, research design, the aim of the study, participant demographics (age, sex, sociodemographic, follow-up), platform, kind of intervention offered, outcomes, result, conclusion, and suggestion. The records were classified into overarching topics to describe the utilization of IoT technology for oral health promotion in children.

## Result

### Search Result

In total, 3,174 articles have been identified from the databases. After removing duplication records and filtering nonrelevant titles and abstracts, 88 full-text articles were retrieved. With the addition of three records from other sources, 91 articles were assessed for eligibility. The reasons for exclusion are displayed at the full-text level in Fig. 1 . Finally, seven articles were included in this study.

### Characteristics of Studies

A total of 602 participants were recorded from the included studies, with 318 males and 284 females. Most participants were selected from dental clinics, either at university clinics (4 studies) 20 24 25 26 or private practices (2 studies). 27 28 Only one study recruited participants from a private school. 29 All studies include United States, 20 Iran, 24 Germany, 27 Netherlands, 28 Saudi Arabia, 25 India, 29 and Belgium. 26

The role of parents in children's oral health maintenance was presented in two studies, 24 29 while the other five studies did not provide the information. Of the seven included articles in the review, an article 29 was quasi-experimental, while the other articles were randomized controlled trail ( Table 1 ).

**Table 1:** The nature of included articles

No	Author, years	Country	Sampel recruitment	The number of participants	Sex		Participation of parents in maintaining oral health	Study design
					Male	Female		
1	Zolfaghariet al 2021 ^24^	Tehran, Iran	The specialty clinic of Tehran School of Dentistry.	58	23	35	Yes	Double-blind, parallel, pretest–posttest, controlled clinical trial
2	Alkilzy et al 2019 ^27^	Germany	Patients from a pediatric dentistry practice in Germany	60	27	33	NA	Controlled, single-blinded, randomized clinical trial
3	Scheerman et al 2020 ^28^	Netherlands	Patients visiting orthodontic clinics in Alkmaar and Leiden, two cities in the Netherlands.	132	59	73	NA	Two-armed, parallel-group, single-blinded randomized controlled trial
4	Alkadhi, et al, 2017 ^25^	Riyadh, Saudi Arabia	At orthodontic clinics of the Riyadh College of Dentistry and Pharmacy, Riyadh, Saudi Arabia.	44	19	25	NA	Two-arm parallel randomized controlled trial
5	Bowen et al 2015 ^20^	Greensburg Pennsylvania, USA	Patients at the Seton Hill University Center for Orthodontics, Greensburg, Pennsylvania	50	21	29	NA	Randomized controlled clinical trial
6	Desai et al 2021 ^29^	Bangalore, India	Private school in Bangalore City, India	247	126	121	Yes	Quasi-experimental
7	Deleuse, et al 2020 ^26^	Belgium	Patients were included from two academic hospitals	38	23	15	NA	Multicenter, randomized, controlled clinical trial

Abbreviation: NA, not available.

### Characteristics of Interventions

The interventions and follow-up periods for the seven publications included in this review are varied ( Table 2 ). There are two studies 24 29 that provided intervention with a gamified mobile-Health (m-Health) application featuring daily push notifications as a form of frequency of intervention. Those in-game applications mostly focus on daily oral hygiene routines. One gives personal advice on how to brush teeth and daily sweets intake based on the questionnaire, with daily evaluation, and the background color of the application will change along with the score obtained from the daily evaluation. 24 Another in-game application used was showing an animated character demonstrating how to brush teeth. The users are expected to follow the instruction along with the song in 3 minutes and evaluate the result using the front camera of the mobile phone to make sure they brush their teeth properly. 29 Meanwhile, two other studies 25 28 presented the participants with the same method as the prior studies without the in-game application. Two studies 26 27 of the m-Health application are tooth-brushing aid apps that provide interactive brushing instructions or are wirelessly connected to electric/manual toothbrushes. However, the study by Bowen et al used a combination of automated text messages reminder system with digital analysis software to measure plaque accumulation. 20 Our research revealed that six of the seven studies investigated dental plaque buildup and gingival health outcomes as indicators of oral hygiene, whereas six of the studies focused on the implementation of m-Health treatments. On the other hand, one research assessed the knowledge and behavior scores on oral health. 28

**Table 2:** Summary of the interventions and outcomes based on specific Internet of Things (IoT) architecture domain

No	Author, years	Follow-up	Aim of the study	Intervention	Outcomes	Result	Conclusion
1	Zolfaghari et al 2021 ^24^	1 month	Assess the efficacy of gamified smartphone application (App) in educating the mothers in terms of their children oral healthcare	Mobile-Health (m-Health)—personalized reminder and scoring system	Dental plaque index (PI) using the “Leo & Silness” modified dental PI	Children with gamified app (intervention group) had better plaque control	Both app (simple app (without game) and gamified app) effectively improved the mothers' oral-health practice and knowledge. The used of gamified app more effective in enhancing children's oral hygiene
2	Alkilzy et al 2019 ^27^	3 months	Evaluating the effect of smartphone application with gravitation sensor in enhancing manual toothbrushing movement	Home healthcare—equipped with gravitation sensor	Dental PI (modified Quingley Hein/QHI) and gingival index (GI), papillary bleeding index (PBI) according to Mühlemann and Saxer [Saxer and Mühlemann, 1975])	The oral health indicator of the intervention group showed better result than control group	The enormous possibilities of a toothbrushing application via the smartphone, at least for medium-term oral hygiene improvement in preschool children and even after excluding the app
3	Scheerman et al 2020 ^28^	3 months	Evaluating the theory-based mobile health application effectiveness in promoting the teenager orthodontic patient	m-Health—equipped with image sensor and followed by telemedicine	Dental PI (Al-Anezi and Harradine PI); and the total number of sites with gingival bleeding (bleeding on marginal probing index). And oral health behavior was measured through a digital questionnaire	The higher decrease of accumulating dental plaque of the intervention group compared to those in control group	A combination of usual care and mobile app utilization provided better assistance to improve the oral hygiene of teenager with fixed orthodontic appliances
4	Alkadhi et al 2017 ^25^	1 month	Comparing the effect of using active reminders in mobile application and verbal instructions in improving oral hygiene	m-Health—giving personalized reminder	Dental PI and GI	Meaningful reduced PI and GI scores of intervention group as to control group	The used of active reminders of oral hygiene instructions via mobile application showed better result than verbal instructions
5	Bowen et al 2015 ^20^	3 months	To evaluate the effect of oral hygiene reminder via text message on plaque removal in orthodontic patients	Electronic-Health (e-Health) and hospital management—equipped with image sensor	Digimizer image analysis software was used to analyze the photographs. Planimetry based assessment of plaque used Digimizer software	Both groups showed significant difference of plaque coverage between baseline and both T1 and T2 as measured using planimetry	Text message reminder is effective to improve plaque removal over 3 months
6	Desai et al 2021 ^29^	1 month	To know the effect of “Brush Up” mobile application on 4-6 years old children oral hygiene behavior in Bangalore	m-Health—equipped with movement sensor	Plaque score using modified visible biofilm index	The intervention group showed better tooth-brushing behavior; marked by a consistently lower PI score during observation period	The use of smart system might improve the learning process of brushing tooth in young children. It also provides the compulsory motivation and reinforcement in plaque control
7	Deleuse et al 2020 ^26^	4.5 months (18 weeks)	To ensure if the use of toothbrush-connected-smartphone application help to boost the adolescent orthodontic patients' compliance	Home healthcare—connecting toothbrush and smartphone, with sensor	PI and GI by the modified Silness and Loe PI, and the presence of White Spot Lesion (WSL)	There were a significant decrease of PI and GI scores in both group. After 12 weeks of observation, the app group showed a significant lower PI scores than control group. There was a remarkable decline of application use over the time	The application showed no effect in oral hygiene promotion

## Discussion

The scoping review found nine research that used IoT as tools or media to support children's oral health, as illustrated in Tables 1 and 2 . In terms of IoT architecture domain (6), most of the studies used m-Health 24 25 28 29 ; while the other two used home healthcare, 26 27 and electronic Health (e-Health). 20 30 Only one study is related to hospital/clinical management. 20 Our review found that IoT is one of the potential tools or media to assist oral health improvement and dental disease prevention in children. IoT helps in building good oral health behavior in children to improve their oral hygiene. Utilizing mobile technology, the Internet, and m-Health application is beneficial in drawing the public's attention; hence, the messages in oral health education and promotion campaigns could reach the desirable audience. 17 31 To sum up, the use of the Internet and mobile apps increases patients' knowledge and awareness to improve oral hygiene. It makes the use of IoT as a dependable method of contact for parents or caregivers in distant locations and compensates for the absence of experienced dental public health practitioners. 32 33 34

An m-Health system denotes a system that provides and supports healthcare services using mobile communication devices, such as smartphones and tablets. It is beneficially effective on both medics and the patients; on one side, it provides healthcare information to medical personnel as well as to the patients. On the other side, it also directly provides healthcare services by means of mobile telemedicine. Meanwhile, IoT is currently progressing rapidly in the Internet by connecting “things” in the physical surroundings of our everyday life. Although m-Health systems development for IoT environments is still in the early stage, the existing research prototypes show the essential potential impact on the healthcare service industry. Since mobile IoT devices, including smartphones and wireless sensors, become covering the essential share in IoT-enabled healthcare services, the role of m-Health is growing in the general concept of e-Health and its evolution to cybermedicine. 35 The essential parts of m-health and IoT are medical devices equipped with sensors and communication devices. 36

Distinguishing between IoT mobile devices and mobile applications in the real world is indeed more difficult compared to those in laboratories. 37 This matter could be the limitation of this study; therefore, we refers to the IoT definition that is an ecosystem of smart objects with sensors, processing and networking technologies integrating and working together to provide an environment in which smart services are taken to the end users. 38 Based on the explanation, the application used in the included studies belongs to IoT since some of them using sensors to evaluate how the users brush their teeth. 26 27 29 Some other studies use sensors that detect plaque accumulation from image taken by the phone's camera 20 followed by private discussion with the expert via telemedicine. 28 One last study sent personalized reminder 25 and messages every night based on the users' answer, and equipped with scoring system. 24

The use of IoT treatments to assess dental plaque buildup and gingival health outcomes as a measure of oral hygiene was the focus of the bulk of the research (6/7). These core findings demonstrate that the safest form of preventive treatment at the moment is mechanical plaque removal at regular intervals. Consequently, tooth-brushing is still a popular and efficient way to clean tooth surfaces. 39 Despite being the most widely used and successful strategy, it is inefficient in young children under the age of 10 owing to a lack of desire and insufficient hand dexterity. Therefore, instructions on brushing the teeth should be given in line with the child's level of preparedness, with assistance from the caregivers involving both systematic education and reinforcement. Despite the requirement for hand agility and proficiency, it is crucial to educate children on oral self-care in line with their psychological development stage. 29

In some studies, 25 26 27 28 a m-Health intervention was given with the main goal of improving oral hygiene habits to reduce dental plaque and gingival bleeding. A notable difference was also found in the plaque index (PI) and gingival index (GI) between the treatment group and control group (GI). Indicators of oral health of the intervention groups were better compared to those in the control groups. In conclusion, using mobile-based health applications has successfully improved children's dental hygiene. The number of smartphone applications has significantly increased recently. The adoption of smartphone applications meant to improve health and hygiene has skyrocketed as a result of the device's rising popularity. 18 2019's app store produced more than 500 dental health-related applications after a thorough search. 40 Previous studies have demonstrated that m-Health may be utilized to enhance oral hygiene, particularly the treatment of gingivitis, and to promote oral health awareness. Additionally, several applications are being created to affect parents' attitudes, beliefs, and actions toward the dental health of their kids. 41

Several studies gamified m-Health applications to intervention group participants, and the findings revealed that the children in the gamified app group had better plaque management. 24 29 According to previous studies, through the facilitation of behavioral change, skill development, and habit formation, gamification in healthcare interventions is a viable strategy for promoting evidence-based dental hygiene practices. 24 29 It may be used to motivate and attract young children to clean their teeth. This is supported by a study stating that the use of an interactive gaming application to monitor kids' compliance with dental hygiene appears to be successful. 2

Although the overall study findings indicated that IoT applications based on smartphone applications are successful in improving children's dental hygiene, the long-term impact should also be examined. The follow-up phase is crucial for experimental research. It is crucial to make sure that interventions are operationalized accordingly (defining what is, and what is not, part of the intervention). 42 It is noteworthy that two 26 27 out of seven studies showed a moderate length of oral hygiene improvement program in preschool children did not draw any significant effect of oral hygiene promotion using the mobile application. Besides, some studies have offered ideas on how to look into a longer follow-up time. 20 25 27 29 30 Hence, the presence review proposes two recommendations for future research. To counteract the anticipated novelty impact, it is first necessary to assess the long-term effect. IoT-based health apps are used for patient education ought to be based on tried-and-true methods of behavior modification. Instructions on how to execute an activity, examples of the desired behavior, reminders and signals, and rewards for displaying the desired behavior should all be provided. However, the second suggestion is the requirement to assess the cost–benefit analysis of using IoT technology to promote oral hygiene.

The development of e-Health and self-management of health issues will profoundly change how healthcare is delivered and information is gathered for the next generation of e-Health solutions. 43 Medical technology linked to the Internet is used to deliver telehealth services including telemonitoring, geriatric supervision, teleconsultations, and robotically assisted surgery. 10 In recent years, dental clinics have started utilizing e-mail (in this case, e-Health) to remind patients of appointments in place of or in addition to phone calls. Patients may now be reminded to brush, floss, maintain oral hygiene, and other chores that primarily seek to avoid dental plaque collection as an indicator of oral hygiene using direct text messaging, which was first used to remind patients of appointments. 20 This study is also limited due to the wide-range of IoT application and devices included in this review, and the limited studies included due to the fact that health system development for IoT environments is in early stage.

## Conclusion

IoT is one of the mediums or instruments that might be used to encourage children's dental health. The most frequently used IoT technology for oral health promotion among children is m-Health, a gamified application that evaluates dental plaque accumulation and gingival health outcomes as an indicator of oral hygiene. The studies suggest that the use of IoT could help in improving oral hygiene and oral health, which further can improve children's oral health. This current review is limited due to the few numbers of studies included. Future research is therefore required to explore long-term effects and assess the cost–benefit analysis of using IoT technology to promote oral health.
