# Supplementary material for: The Use of Internet of Things (IoT) Technology to Promote Children's Oral Health: A Scoping Review
Source: Eur J Dent. 2024 Jan 10;18(3):703–11. doi: 10.1055/s-0043-1776116 (PMC11290912; doi:10.1055/s-0043-1776116)
Supplement: Supplementary file 1 — Supplementary Material [file 10-1055-s-0043-1776116-s2322690.pdf]

Appendix 1

The Following Search Method Was Used in PubMed

((((((((((((("Dental Care for Children/epidemiology"[Mesh] OR "Dental Care for Children/instrumentation"[Mesh] OR "Dental Care for Children/methods"[Mesh] OR "Dental Care for Children/therapeutic use"[Mesh] OR "Dental Care for Children/therapy"[Mesh])) OR "Computer Storage Devices"[Mesh]) OR "Instructional Film and Video") OR "Telemedicine"[Mesh]) OR ("Medical Informatics Applications/ instrumentation"[Mesh] OR "Medical Informatics Applications/ methods"[Mesh] OR "Medical Informatics Applications/ therapy"[Mesh])) OR "Internet of Things"[Mesh]) OR ("Internet/instrumentation"[Mesh] OR "Internet/trends"[Mesh])) OR ("Social Media/instrumentation"[Mesh] OR "Social Media/trends"[Mesh])) OR "Reminder Systems/instrumentation"[Mesh]) OR "Electronic Mail/instrumentation"[Mesh]) OR "Mobile Applications"[Mesh]) AND "Oral Health"[Mesh]) OR "Health Knowledge, Attitudes, Practice"[Mesh]) OR ("Dental Caries/diagnosis"[Mesh] OR "Dental Caries/diagnostic imaging"[Mesh] OR "Dental Caries/diet therapy"[Mesh] OR "Dental Caries/drug effects"[Mesh] OR "Dental Caries/drug therapy"[Mesh] OR "Dental Caries/instrumentation"[Mesh] OR "Dental Caries/prevention and control"[Mesh] OR "Dental Caries/therapy"[Mesh])

The Following Search Method Was Used in Ebscohost

"Dental Care for Children" AND "Computer Storage" OR "Instructional Film and Video" OR Telemedicine OR Telehealth OR Telecare OR "Medical Informatics Applications" OR "Internet of Things" OR Internet OR "Social Media" OR "Reminder Systems" OR "Electronic Mail" OR email OR "Mobile Applications" OR apps OR "mobile apps" OR mhealth OR ehealth OR "cell phone" OR "online intervention" OR "online therapy" OR "computer assisted therapy" AND "Oral Health" OR "oral hygiene" OR "dental health" OR "mouth care" OR "oral care" OR "oral health behavior" OR "Health Knowledge, Attitudes, Practice" OR "Dental Caries" OR "dental decay" OR "dental cavity" OR "dental cavities" OR "tooth decay" OR "early childhood caries"

The Following Search Method Was Used in Scopus

"Dental Care for Children" AND "Computer Storage" OR "Instructional Film and Video" OR Telemedicine OR Telehealth OR Telecare OR "Medical Informatics Applications" OR "Internet of Things" OR Internet OR "Social Media" OR "Reminder Systems" OR "Electronic Mail" OR email OR "Mobile Applications" OR apps OR "mobile apps" OR mhealth OR ehealth OR "cell phone" OR "online intervention" OR "online therapy" OR "computer assisted therapy" AND "Oral Health" OR "oral hygiene" OR "dental health" OR "mouth care" OR "oral care" OR "oral health behavior" OR "Health Knowledge, Attitudes, Practice" OR "Dental Caries" OR "dental decay" OR "dental cavity" OR "dental cavities" OR "tooth decay" OR "early childhood caries"

 © 2024. The Author(s).
